# The neuromuscular system of *Pycnophyes kielensis* (Kinorhyncha: Allomalorhagida) investigated by confocal laser scanning microscopy

**DOI:** 10.1186/s13227-016-0062-6

**Published:** 2016-11-28

**Authors:** Andreas Altenburger

**Affiliations:** Section for Evolutionary Genomics, Natural History Museum of Denmark, University of Copenhagen, Sølvgade 83, 1307 Copenhagen, Denmark

**Keywords:** Ecdysozoa, Kinorhyncha, Segmentation, Evolution, Nervous system, Muscular system

## Abstract

**Background:**

Kinorhynchs are ecdysozoan animals with a phylogenetic position close to priapulids and loriciferans. To understand the nature of segmentation within Kinorhyncha and to infer a probable ancestry of segmentation within the last common ancestor of Ecdysozoa, the musculature and the nervous system of the allomalorhagid kinorhynch *Pycnophyes kielensis* were investigated by use of immunohistochemistry, confocal laser scanning microscopy, and 3D reconstruction software.

**Results:**

The kinorhynch body plan comprises 11 trunk segments. Trunk musculature consists of paired ventral and dorsal longitudinal muscles in segments 1–10 as well as dorsoventral muscles in segments 1–11. Dorsal and ventral longitudinal muscles insert on apodemes of the cuticle inside the animal within each segment. Strands of longitudinal musculature extend over segment borders in segments 1–6. In segments 7–10, the trunk musculature is confined to the segments. Musculature of the digestive system comprises a strong pharyngeal bulb with attached mouth cone muscles as well as pharyngeal bulb protractors and retractors. The musculature of the digestive system shows no sign of segmentation. Judged by the size of the pharyngeal bulb protractors and retractors, the pharyngeal bulb, as well as the introvert, is moved passively by internal pressure caused by concerted action of the dorsoventral muscles. The nervous system comprises a neuropil ring anterior to the pharyngeal bulb. Associated with the neuropil ring are flask-shaped serotonergic somata extending anteriorly and posteriorly. A ventral nerve cord is connected to the neuropil ring and runs toward the anterior until an attachment point in segment 1, and from there toward the posterior with one ganglion in segment 6.

**Conclusions:**

Segmentation within Kinorhyncha likely evolved from an unsegmented ancestor. This conclusion is supported by continuous trunk musculature in the anterior segments 1–6, continuous pharyngeal bulb protractors and retractors throughout the anterior segments, no sign of segmentation within the digestive system, and the absence of ganglia in most segments. The musculature shows evidence of segmentation that fit the definition of an anteroposteriorly repeated body unit only in segments 7–10.

**Electronic supplementary material:**

The online version of this article (doi:10.1186/s13227-016-0062-6) contains supplementary material, which is available to authorized users.

## Background

### Kinorhyncha

Kinorynchs are microscopic, meiobenthic, marine invertebrates, generally less than 1 mm in length [1]. They are ecdysozoan animals grouped together with unsegmented Priapulida and Loricifera in the clade Scalidophora, which, together with Nematoidea (Nematoda and Nematomorpha), constitutes the Cycloneuralia [2]. So far, there is no clear indication that the Cycloneuralia is a monophyletic group [3–7].

Phylogenetically, kinorhynchs are divided into two major clades which are called Cyclorhagida and Homalorhagida or Allomalorhagida [8, 9]. The body plan of kinorhynchs comprises an eversible head (introvert) with several rings of scalids, a protrusible mouth cone, a neck, and a trunk with 11 segments (called zonites in older literature) [10, 11]. Kinorhynchs use their introvert to move in between sand grains on the sea floor and to feed on bacteria [12, 13]. The musculature and nervous system of members of the cyclorhagid kinorhynch genus *Echinoderes* have been investigated previously [14, 15]. These studies found a combination of segmented and unsegmented features in the myoanatomy and nervous system of kinorhynchs.

### Segmentation

Segmentation is the anteroposterior repetition of body units and an important feature of many bilaterian animals [16, 17]. The importance of segmentation as a phylogenetic character is controversial. The traditional view was that the segmented annelids and arthropods are closely related [18]. This hypothesis has been challenged by phylogenetic analyses based on molecular data and by the fact that it is difficult to clearly define segmentation [19, 20]. Currently, there is no consensus whether segmentation evolved once in the early history of metazoans or non-homologous segmentation evolved several times independently in various metazoan lineages [21].

The presence of true segmentation defined by substructures of segments such as muscles and nephridia in specific spatial patterns in the distantly related Annelida and Panarthropoda has led to the hypothesis that the last common ancestor of Bilateria must have been complex and segmented [22–25]. The main argument for this hypothesis is the complexity involved in generating a segmented body plan [26]. A common origin of segmentation across the Bilateria implies a common mechanism underlying the process of segmentation in disparate segmented animals such as vertebrates, annelids, and arthropods. Such a mechanism has been proposed to be a segmentation ‘clock’ of oscillation gene expression involving Notch pathway components [27]. A segmentation ‘clock,’ however, is not present in all investigated animals. *Drosophila melanogaster* for example has a distinct mode of segmentation where maternal, gap, pair rule and segment polarity genes organize the simultaneous formation of segments [28]. Also, the underlying mechanisms that utilize the conserved roles of genes involved in segment formation might not be identical [29]. A common origin of segmentation across the Bilateria would mean that unsegmented animals must have lost segmentation. Kinorhynchs are interesting animals to study with respect to the ancestry of segmentation, because they show an exteriorly segmented body plan and are members of the superphylum Ecdysozoa that includes segmented phyla such as arthropods and onychophorans and unsegmented phyla such as nematodes.

### Musculature

The musculature of several kinorhynch species has been described based on light microscopy [9, 12, 30–32] and transmission electron microscopy [1, 31, 33, 34]. The musculature of *P. kielensis* has been investigated by phalloidin staining and confocal laser scanning microscopy [35, 36]. However, due to further development of confocal microscopes, the present study goes into greater detail to resolve muscular structures related to the introvert which could not previously be resolved. Using immunostainings and confocal laser scanning microscopy, musculature has already been studied in the cyclorhagids *Antygomonas* sp. and members of *Echinoderes* [14, 37].

Kinorhynch trunk musculature comprises longitudinal and dorsoventral muscles [9]. The longitudinal muscles are attached to thickenings of the cuticle which are present at the segment borders and called pachycycli. The longitudinal muscles run ventrally and dorsally in pairs [9]. One pair of dorsoventral muscles runs between the sternal (ventral) and tergal (dorsal) trunk plate in each segment [9]. Cyclorhagids have additionally lateral oblique muscles between two subsequent segments, or between the tergal plates of subsequent segments. The introvert region comprises circular muscles at the base of the scalids of rings 06 and 07. There are 10 (Allomalorhagida) or 16 (Cyclorhagida) head retractor muscles between the base of the head scalids of rings 05 and 06 and posterior trunk segments. The mouth cone comprises mouth cone retractor muscles between the base of the mouth cone and posterior segments, as well as pharynx protractor muscles between the base of the mouth cone and the posterior end of the pharynx. The placids in the neck region are connected by circular muscles, and the posterior part of the male gonads is surrounded by a muscle net [9]. Several muscles are associated with the digestive system such as circular and longitudinal muscles in the mouth cone, pharyngeal ring and radial muscles, longitudinal and circular muscles around the midgut, and dilatator muscles at the hindgut [12, 31, 33, 36, 37]. In juveniles, the longitudinal musculature is continuous between the segments, whereas in adults the musculature visible by f-actin staining is separated in each trunk segment [35, 36]. The trunk musculature is used to move the body ventrally, dorsally and laterally, whereas the pharynx protractors and mouth cone retractors are responsible for the back and forth movement of the introvert [12]. Circular muscles serve to close the trunk anteriorly, once the introvert is retracted completely [31].

To add further information to the available data, the musculature of the allomalorhagid kinorhynch species *Pycnophyes kielensis* was investigated*. P. kielensis* is known from the Bay of Kiel, Germany; the Øresund close to Vedbæk, Denmark; the Greifswalder Bodden, Germany; and the North Sea around Helgoland and Sylt, Germany [12, 38–40].

### Nervous system

Understanding the evolution of nervous systems requires a detailed morphological investigation of all bilaterian groups, especially those at informative nodes of phylogenies [16, 41]. Comparable to the controversy about segmentation in the last common ancestor (LCA) of Bilateria, there is an ongoing discussion about the complexity of the nervous system in this LCA. It is often assumed that complex central nervous systems (CNS) that are present in most higher metazoan animals can be traced back through evolution to a nerve net cnidarian-like ancestor [42]. What the nervous system of the LCA of Bilateria looked like is, however, controversial. Some favor a several times independent evolution of a complex CNS from a bilaterian LCA with a nerve net [43–45], and others argue for a LCA with a complex CNS and subsequent loss of complexity in brainless Bilateria [22, 42, 46–53]. It appears that much of the genetic machinery necessary for a nervous system was present in the ancestor of all extant animals [54].

Kinorhynch nervous systems have been investigated using TEM in *Pycnophyes kielensis*, *Pycnophyes beaufortensis, Pycnophyes greenlandicus, Echinoderes aquilonius, Echinoderes asiaticus, Echinoderes capitatus* [1, 31, 33, 55, 56], and immunostainings together with confocal laser scanning microscopy in the cyclorhagids *Echinoderes spinifurca*, *Antygomonas paulae*, and *Zelinkaderes brightae* [15].

The nervous system is intraepidermal, with a circumpharyngeal brain which is divided into the three areas, frontal to caudal: somata–neuropil–somata. Somata are the bulbous ends of neuron cells, and the neuropil is a cluster of neurites in which neuronal somata do not occur [57]. Attached to the brain is a ventral nerve cord with lateral and ventral somata, additional longitudinal nerve cords, and sensory spots in the trunk area [15, 31, 33]. Other studies found the forebrain to be 10-lobed with numerous somata (perikarya), a midbrain with few somata but abundant neuropile, and a hindbrain with numerous somata [1, 58]. In this study, the longitudinal nerve cords in the trunk are connected by two commissures per trunk segment [58]. Besides their locomotory function, the scalids are also innervated sensory organs [55].

The nervous system organization with a circumpharyngeal brain and somata–neuropil–somata pattern is used as a uniting character for Nematoda, Nematomorpha, Priapulida, Kinorhyncha, and Loricifera forming the clade Cycloneuralia [59]. The data available so far is mainly based on investigations with transmission electron microscopes. This data will be amended herein with a description of the immunoreactive nervous system by antibody stainings in combination with confocal laser scanning microscopy and 3D reconstruction software.

## Methods

### Specimen collection and fixation

Specimens were collected from intertidal mud on the German island Sylt in the vicinity of Braderup (N 54°56′03.7″, E 08°21′39.0″) at low tide on the following dates 14.03.2012, 5.6.2012, and 22.05.2013. The area is covered by very fine mud. The mud surface was collected to a depth of approximately 1 cm and then brought to the wet laboratory at the Alfred-Wegener Wadden Sea Station Sylt. There, specimens of *P. kielensis* were extracted using the bubble and blot method [60]. Animals were sorted under a dissecting microscope and fixed for 1 h at room temperature in 4% paraformaldehyde in 0.1 M phosphate buffer (PB). Then, the specimens were washed three times for 15 min each in 0.1 M PB and stored in 0.1 M PB containing 0.1% NaN_3_ at 4 °C.

### Immunostainings and confocal laser scanning microscopy (CLSM)

For f-actin stainings, specimens were cut with a scalpel at one side of the trunk to allow free movement of molecules into the tissue. Subsequently, specimens were washed three times for 15 min in 0.1 M PB at room temperature and left in 0.1 M PB containing 0.2% Triton X-100 (Sigma-Aldrich, St. Louis, MO, USA) for 1 h in order to permeabilize the tissue. Specimens were then transferred into a solution of 0.1 M PB containing 0.2% Triton X-100 and 1:20 diluted Alexa Fluor 488 phalloidin (Thermo Fisher Scientific, Molecular Probes, Eugene, OR, USA). Specimens were left in this solution for 72 h at 4 °C and subsequently washed three times for 15 min in 0.1 M PB. Finally, the specimens were embedded on glass slides using Fluoromount G (SouthernBiotech, Birmingham, AL, USA).

For stainings of the nervous system, specimens were cut in the center with a scalpel to allow for free diffusion of antibodies and incubated overnight at 4 °C in 6% normal goat serum (Acris Antibodies GmbH, Herford, Germany) in 0.1 M PB and 0.2% Triton X-100 (blocking solution). Subsequently, specimens were incubated for 72 h at 4 °C in blocking solution containing a mixture of either a 1:400 diluted primary serotonin antibody (rabbit, ImmunoStar, Hudson, WI, cat. # 20080), or a 1:400 diluted FMRFamide antibody (rabbit, BioTrend cat. # FA 1155-0100), together with 1:400 diluted acetylated alpha tubulin antibodies (mouse, monoclonal, Sigma, cat. # T-7451) and anti-tyrosinated alpha tubulin (mouse, Sigma, cat. # T9028). Subsequently, specimens were washed in the blocking solution overnight at 4 °C with four changes in the blocking solution. Then, the secondary antibodies [either FITC goat anti-rabbit (Sigma, cat. #F9887), Alexa 594 goat anti-rabbit (Thermo Fisher Scientific, cat# A-11037), or Alexa Fluor 633 goat anti-rabbit (Thermo Fisher Scientific cat. # A21070) and TRITC goat anti-mouse (Sigma, cat. #T5393) or Alexa 488 goat anti-mouse (Thermo Fisher Scientific cat. # A11001)] were added in a 1:300 dilution in the blocking solution, and the samples were incubated for 48 h. Finally, the specimens were washed three times for 15 min each in 0.1 M PB and embedded in Fluoromount G on glass slides.

Negative controls omitting either the phalloidin dye or the respective secondary antibody were performed in order to test for signal specificity and rendered no signal.

### Scanning electron microscopy (SEM)

For SEM, specimens were dehydrated using a graded series of ethanol, transferred to acetone and critical point dried. Dried specimens were mounted on aluminum stubs, sputter coated with a platinum/palladium mix, and examined with a JEOL JSM-6335F field emission SEM (JEOL GmbH, Eching, Germany).

### Image handling and processing

Figure plates were assembled using Creative Suite 6 (Adobe, San Jose, California, USA). To obtain fine detail micrographs of entire specimens, maximum or average projections of three confocal scans done on the same day of the same specimen (one anterior, one central, and one posterior), using identical settings on the microscope, were merged using the Photoshop ‘photomerge’ feature with ‘auto’ settings.

Transverse projections were done with ImageJ (IJ 1.46r) [61]. 3D reconstructions were done with Imaris 5.7.2 (Bitplane, Belfast, UK).

## Results

### SEM and outer morphology

**Fig. 1:**
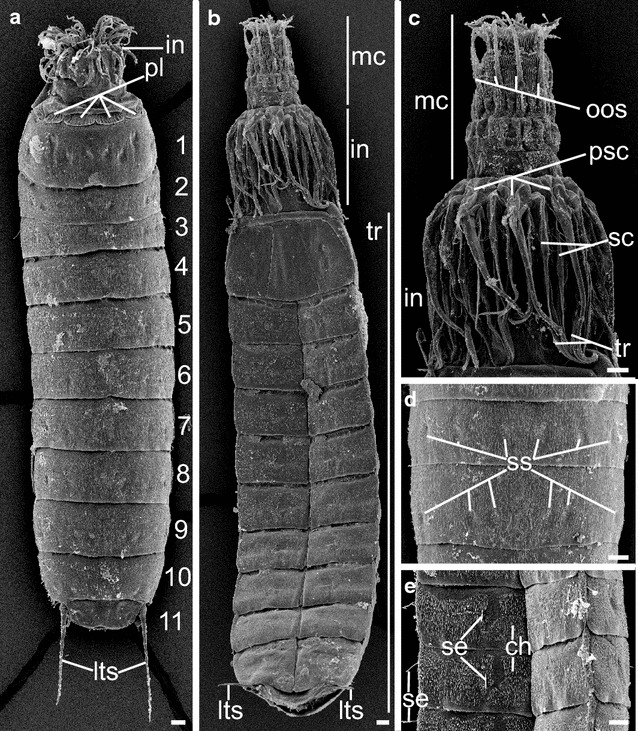
External morphology of *Pycnophyes kielensis*. SEM micrographs, specimens are oriented with the anterior end upwards, *numbers* indicate segment numbers and the *scale bars* equal 10 µm. **a** Dorsal view of *P. kielensis* with four dorsal placids, partly everted introvert, eleven trunk segments, and lateral terminal spines. **b** Ventral view of a female with everted mouth cone and the scalid bearing introvert, 11 segments of the trunk and lateral terminal spines. **c** Mouth cone with outer oral styles, introvert with primary spinoscalids of the primary scalid ring, spinoscalids of the subsequent rings, as well as trichoscalids of ring 07. **d** Dorsal view of segments 8 and 9 with sensory spots. **e** Ventral view of segments 3 and 4 with cuticular hairs and setae in lateral and ventral position. *ch* cuticular hairs, *in* introvert, *lts* lateral terminal spine, *mc* mouth cone, *oos* outer oral styles, *pl* placids, *psc* primary spinoscalids, *sc* scalids; *se* setae, *ss* sensory spot, *tr* trichoscalid

*Pycnophyes kielensis* is a typical allomalorhagid kinorhynch with triangular cross section, introvert, neck, 11 trunk segments and two lateroterminal spines (Fig. 1a). Segment 1 consists of one tergal plate, two episternal plates and a midsternal plate; segments 2–11 comprise one tergal and two sternal plates (Fig. 1a, b). As all kinorhynchs, *P. kielensis* moves by everting the introvert (Fig. 1c; Additional file 1). When retracted, the introvert is covered by eight placids (neck plates) (Fig. 1a). The mouth cone has longitudinal cuticular rods and nine non-articulated outer oral styles (Fig. 1c). The introvert has pentaradial symmetrically arranged scalids that are organized in seven rings (Fig. 1c). Ring 01 contains the primary spinoscalids, rings 02–06 the spinoscalids, and ring 07 the trichoscalids (Fig. 1c). The trunk is covered by cuticular hairs, setae, and sensory spots on the dorsal and the ventral side (Fig. 1d, e).

### Musculature

#### Anterior part with introvert, mouth cone and pharyngeal bulb

**Fig. 2:**
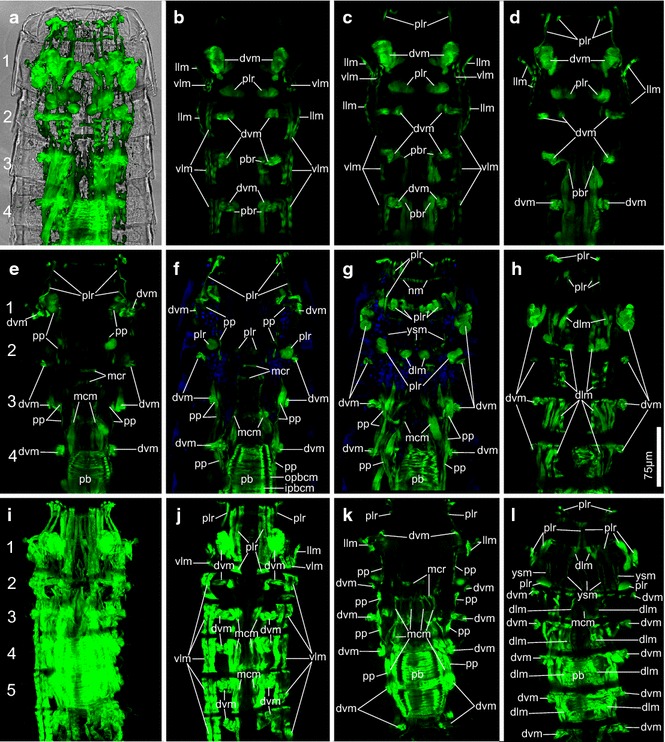
Musculature of *Pycnophyes kielensis*; anterior with introvert inverted. Micrographs obtained with a confocal laser scanning microscope of two kinorhynchs with introvert slightly everted. The musculature was stained with Alexa-488-labeled phalloidin and appears *green*. **a** and **i** are maximum projections, **b**–**h** are single scans from the maximum projection shown in A from ventral to dorsal. **j**–**l** are single scans from the maximum projection shown in I from ventral to dorsal. *Numbers* indicate segment number, the animals are oriented with the anterior end upwards, and the *scale bar* is the same for all micrographs. **a** *P. kielensis* with overlay of a musculature over a transmission image to show the position of the muscles relative to the cuticle. **b** Ventral scan with ventral longitudinal muscles. **c** Ventral scan slightly more dorsal than **b** showing the lateral longitudinal muscles between segment 1 and 2. **d** Ventral view slightly more dorsal than **c** showing the insertion of the lateral longitudinal muscles on the cuticle in segment 1. **e** Ventral view slightly more dorsal than **d** showing the pharyngeal bulb and attached mouth cone muscles. **f** Dorsal view slightly more dorsal than **e** with pharyngeal bulb protractor muscles and inner and outer pharyngeal bulb circular muscle layers. **g** Dorsal view slightly more dorsal than **f** with placid retractors and DAPI staining in *blue* to show the position of the brain. **h** Dorsal view with dorsal longitudinal musculature. **i** Maximum projection of a specimen with partly everted introvert. **j** Ventral scan with complete placid retractor muscles. **k** Longitudinal scan with the mouse cone musculature that is connected to the pharyngeal bulb and overlain by pharyngeal bulb protractor muscles. **i** Dorsal scan with view on the scalid muscles. *dlm* dorsal longitudinal muscle, *dvm* dorsorventral muscle, *ipbcm* inner pharyngeal bulb circular muscle, *llm* lateral longitudinal muscle, *mcr* mouth cone ring muscle, *nm* neck muscle, *opbcm* outer pharyngeal bulb circular muscle, *mcm* mouth cone muscle, *pb* pharyngeal bulb, *pbr* pharyngeal bulb retractor, *plr* placid retractor, *pp* pharyngeal bulb protractor, *ysm* y-shaped muscle, *vlm* ventral longitudinal muscle

**Fig. 3:**
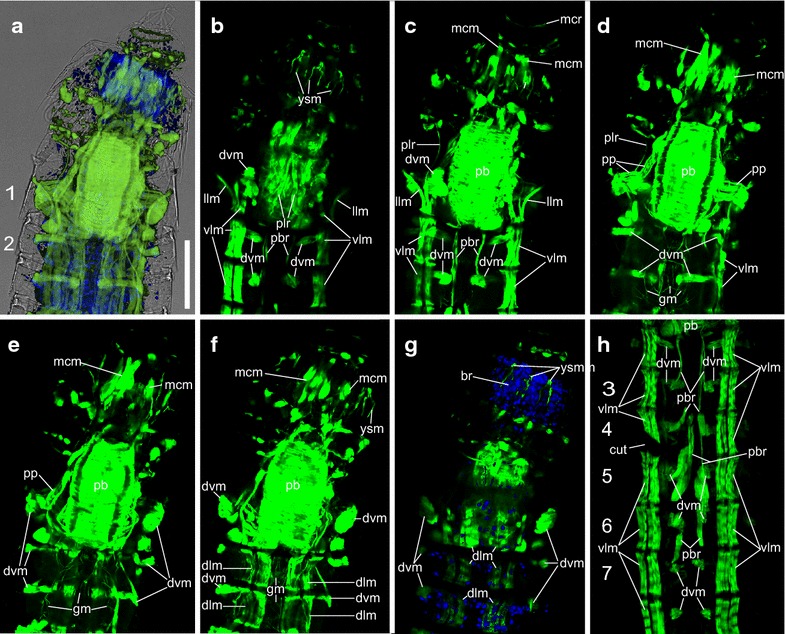
Musculature of *Pycnophyes kielensis*; anterior with introvert everted. Micrographs obtained with a confocal laser scanning microscope of one kinorhynch with introvert fully everted. The musculature was stained with Alexa-488-labeled phalloidin and appears *green*. **a** is an average projection, **b**–**g** are single scans from the average projection shown in **a** from ventral to dorsal. *Numbers* indicate segment number, the animal is oriented with the anterior end upwards, the *scale bar* is valid for all micrographs and represents 75 µm. **a** Average projection overlay over a transmission micrograph to show the position of the muscles within the cuticle. **b** Ventral most view with placid retractors and primary spinoscalid musculature. **c** Slightly more dorsal view than **b** with pharyngeal bulb retractors. **d** Slightly more dorsal view than **c** with pharyngeal bulb protractors, gut musculature and mouth cone muscles. **e** Slightly more dorsal view than **d** with pharyngeal bulb protractors inserting at the same position as the dorsoventral muscles in segment 1. **f** Slightly more dorsal view than **e** with dorsal longitudinal muscles. **g** Slightly more dorsal view than **f** with y-shaped muscles on the dorsal side and DAPI staining of the brain in *blue*. **h** Maximum projection of the trunk of another specimen showing the insertion points of the pharyngeal bulb retractors in segments 4, 5, and 6. *br* brain, *dlm* dorsal longitudinal muscle, *dvm* dorsoventral muscle, *gm* gut musculature, *llm* lateral longitudinal muscles, *mcm* mouth cone muscle, *mcr* mouth cone ring muscle, *pb* pharyngeal bulb, *pbr* pharyngeal bulb retractor, *plr* placid retractor, *pp* pharyngeal bulb protractor, *ysm* y-shaped muscle, *vlm* ventral longitudinal muscle

The anterior part of *P. kielensis* includes the introvert, mouth cone, pharyngeal bulb, and the placid closing apparatus. Segments 1–4 have dorsovental muscles, ventral longitudinal muscles as well as dorsal longitudinal muscles (Fig. 2a–l). Between segments 1 and 2 are additional lateral longitudinal muscles that insert together with the ventral longitudinal muscles in segment 2 and attach laterally to the cuticle in segment 1 (Figs. 2b–d, j, k, 3b, c). When the introvert is inverted, the pharyngeal bulb has its position between segments 4 and 6 (Fig. 2a, i, 4a). When the introvert is everted, the pharyngeal bulb is positioned anterior to segment 2 (Fig. 3a). The pharyngeal bulb consists of two layers of circular musculature: inner and outer pharyngeal bulb circular muscles (Fig. 2f). On the outside interconnected with the pharyngeal bulb are nine mouth cone muscles which run from the approximate center of the pharyngeal bulb toward the anterior, and end in a mouth cone ring muscle. An additional mouth cone ring muscle is present at the anterior tip of the mouth cone (Figs. 2e, f, k, 3c). Interconnected to the pharyngeal bulb on the lateral side are two pharyngeal bulb protractor muscles that attach dorsally and ventrally to the cuticle in segment 1 (Figs. 2f, k, 3d, e). The attachment of the pharyngeal bulb protractor muscles in segment 1 is at the same position as the dorsoventral muscles within this segment. Posteriorly, the pharyngeal bulb has pharyngeal bulb retractors that attach together with the dorsoventral musculature to the ventral cuticle in segments 3, 4, 5, and 6 (Fig. 3h).

**Fig. 4:**
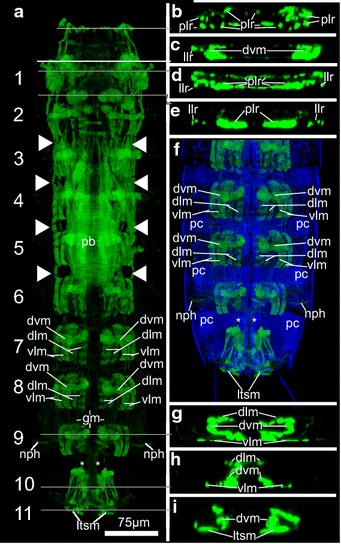
Musculature of the trunk of *Pycnophyes kielensis*. Micrographs obtained with a confocal laser scanning microscope of one specimen with introvert inverted. The musculature was stained with Alexa-488-labeled phalloidin and appears *green*. Cell nuclei where stained with DAPI and appear *blue* together with autofluorescence of the cuticle. **a** and **f** are maximum projections, **b**–**e** and **g**–**i** are transverse projection of the maximum projection shown in **a** from anterior to posterior. *Numbers* indicate segment number, the animals are oriented with the anterior end upwards, and the *scale bar* is the same for all micrographs. **a** Overview showing the discrete sets of dorsal and ventral longitudinal as well as dorsoventral muscles in segments 7–9 and the continuous muscle fibers running over segment borders in segments 1–6 (*arrowheads*). **b** Transverse section of the anterior end of the specimen with eight paired placid retractors. **c** Transverse section of segment one with dorsoventral muscles and lateral longitudinal muscle. **d** Transverse section of segment one with placid retractor muscles. **e** Transverse section of segment 1 close to segment 2 with attachment site of the ventral placid retractors to the cuticle. **f** Posterior part of the specimen with pachycycli as attachment points for the longitudinal musculature in segments 7–10. **g** Transverse section of segment 9 with dorsal longitudinal musculature, ventral longitudinal musculature, and dorsoventral musculature. **h** Transverse section of segment 10 with dorsoventral musculature. **i** Transverse section of segment 11 with lateral terminal spine muscles. *arrowheads* points where ventral longitudinal musculature runs over segment borders, *dlm* dorsal longitudinal muscle, *dvm* dorsoventral muscle, *gm* gut musculature, *llr* lateral longitudinal muscles, *ltsm* lateral terminal spine muscle, *nph* nephridia, *pc* pachycycli, *plr* placid retractors, *vlm* ventral longitudinal muscle

**Fig. 5:**
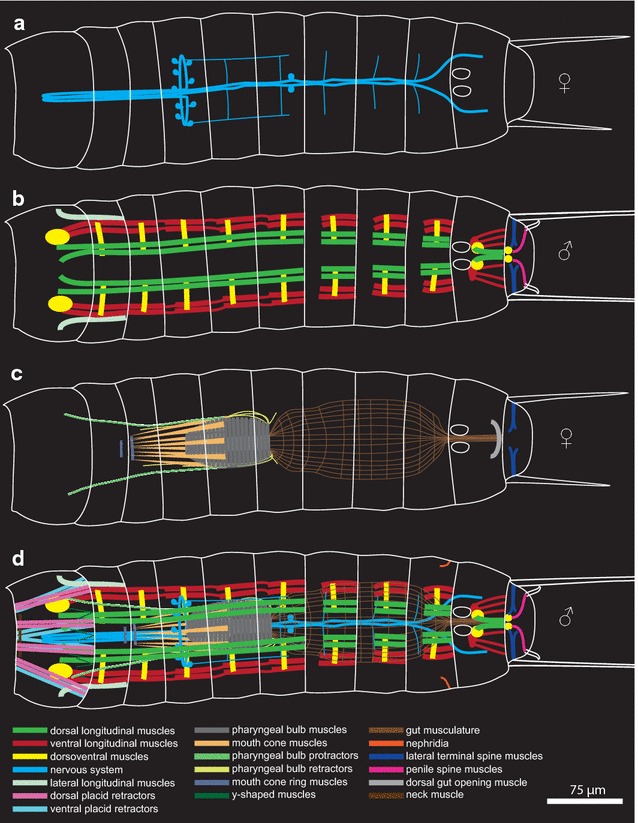
Schematic drawing of the musculature and nervous system in *Pycnophyes kielensis*. **a** Nervous system. **b** Trunk musculature and male musculature in segment 11. **c** Musculature of the digestive system and female musculature in segment 10–11. **d** Complete musculature and nervous system in a male

Further prominent muscles of the anterior part are the eight paired placid retractors in segment 1 which run from the pachycyclus between segments 1 and 2 to the placids at the anterior end of segment 1 and form the closing apparatus when the introvert is inverted (Figs. 2a, c–l, 4b, e). On the anterior dorsal side of segment 1 runs one transversal neck muscle (Figs. 2g, 5d). In between the primary spinoscalids are eight y-shaped muscles that are situated at the base of segment 1 when the introvert is inverted (Fig. 2g, l). When the introvert is everted, the y-shaped muscles are upside down (ʎ-shaped), and positioned anterior of segment 1 within the brain (Fig. 3g). There is no visible connection between the y-shaped muscles and other muscles in *P. kielensis.*

### Trunk

The trunk musculature of *P. kielensis* consists of ventral and dorsal longitudinal muscles in segments 1–10, as well as dorsoventral muscles in segments 1–11 (Figs. 4a–i, 5b). The dorsal and ventral longitudinal muscles are present in pairs, symmetrically on each side of the segments in segment 2–9 (Fig. 4a). The organization is different in segment 10 (see below). The dorsoventral muscles run from midventral to dorsolateral in the center of segments 2–9, from midventral to middorsal in segments 10–11, and from lateroventral to laterodorsal in segment 1 (Figs. 4c, h, i, 5b). The ventral and dorsal longitudinal muscles insert on the cuticle inside of the animal at apodemes like midventral thickenings on the ventral side of segment 10 and pachycycli on the ventral and dorsal side of segments 2–10 (Fig. 4f). In segment 1, the dorsal and ventral longitudinal musculature inserts at the posterior pachycyclus, in the center of the segment. Segments 7–9 have an isolated set of ventral and dorsal longitudinal muscles as well as dorsoventral muscles without any muscular connection running over the segment borders (Fig. 4a, f). Between segments 1–6, a strand of ventral and dorsal longitudinal musculature runs over the segment borders formed by the pachycycli (Fig. 4a).

### Posterior part with segments 10 and 11

**Fig. 6:**
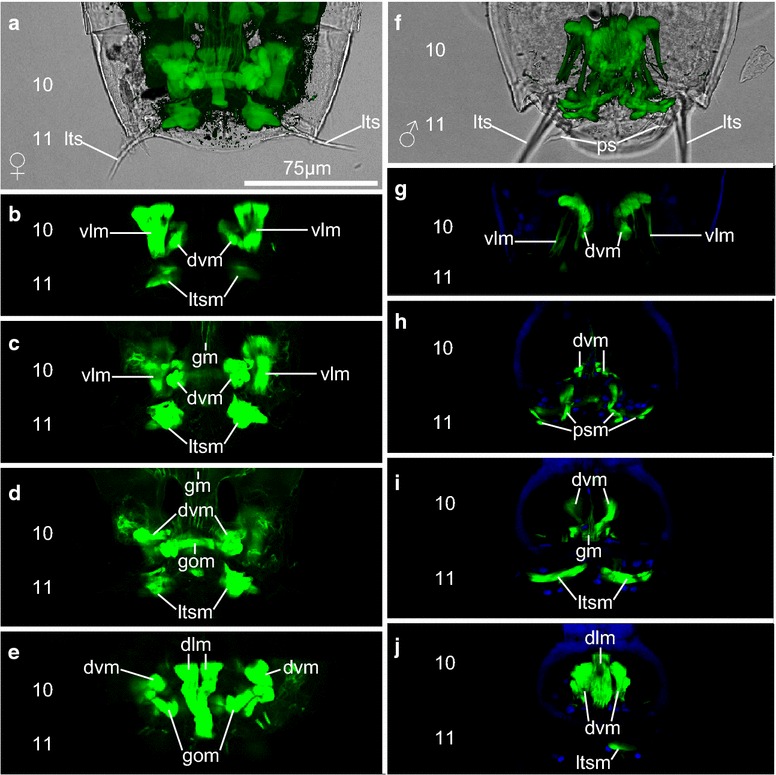
Musculature of the posterior segments of *Pycnophyes kielensis*. Micrographs obtained with a confocal laser scanning microscope of two specimens, one female and one male. The musculature was stained with Alexa-488-labeled phalloidin and appears *green*. Cell nuclei where stained with DAPI and appear *blue* together with autofluorescence of the cuticle. **a** and **f** are maximum projections, **b**–**e** and **g**–**j** are single scans from of the maximum projections shown in **a** and **f**, respectively, from ventral to dorsal. *Numbers* indicate segment number, the animals are oriented with the anterior end upwards, and the *scale bar* is the same for all micrographs. **a** Female with overlay of a maximum projection of musculature over a transmission scan to show the relative position of the musculature within the cuticle and in relation to the lateral terminal spines. **b** Ventral scan of **a** with ventral longitudinal and lateral terminal spine muscles. **c** Ventral scan slightly more dorsal than **b** with prominent lateral terminal spine muscles. **d** Dorsal scan slightly more dorsal than **c** with dorsoventral muscles and gut opening muscle. **e** Dorsal scan with dorsal longitudinal muscles and the dorsal insertion point of dorsoventral muscles. **f** Male with overlay of maximum projection of musculature over transparent scan to show the relative position of the musculature to the cuticle, the penile spines, and the lateral terminal spines. **g** Ventral view with ventral longitudinal muscles and insertion point of dorsoventral muscles to the cuticle. **h** Ventral view slightly more dorsal than **g** with penile spine muscles. **i** Dorsal view slightly more dorsal than **h** with lateral terminal spine muscles. **j** Dorsal view with dorsal longitudinal muscles. *dlm* dorsal longitudinal muscle, *dvm* dorsoventral muscle, *gm* gut musculature, *gom* gut opening muscle, *lts* lateral terminal spine, *ltsm* lateral terminal spine muscle, *psm* penile spine muscle, *vlm* ventral longitudinal muscle

In the posterior part of *P. kielensis* are the gut, nephridia, and the reproductive organs. The musculature of the gut consists of a net of longitudinal and circular muscles (Figs. 4a, 5c). The gut is flexible and moves together with the pharyngeal bulb when the introvert is everted. Depending on the position of the pharyngeal bulb, the gut extends between segments 2 and 10 when the introvert is everted or 6 and 10 when the introvert is inverted (Figs. 3d, 4a). The gut opens posteriorly to the environment between segments 10 and 11 (Fig. 6d, i). The nephridia do not contain musculature, but numerous microvilli that contain f-actin and are thus visible by f-actin stainings in segment 9 (Fig. 4a). The musculature in segment 10 and 11 shows some differences between males and females. Both male and females have a set of ventral longitudinal, dorsoventral, and dorsal longitudinal muscles in segment 10 (Fig. 6a–j). The ventral longitudinal muscles start out paired at the anterior pachycyclus and merge toward one strand posteriorly (Fig. 6b, g). The dorsal longitudinal muscle in segment 10 consists of only one pair that starts at the anterior end and merges to a single strand in the posterior end of the segment (Fig. 6e, j). Both females and males have prominent lateral terminal spine muscles in segment 11, to move the lateral terminal spines (Fig. 6b–d, i, j). Differences in the musculature between males and females are a dorsal gut opening muscle in segment 10 which is only present in females (Fig. 6d), and the penile spine muscles in segment 11 which are only present in males (Fig. 6h). There is no musculature around the gonads in males or females.

### Nervous system

**Fig. 7:**
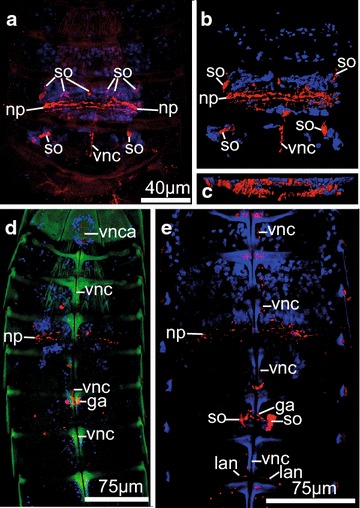
Serotonergic nervous system of *Pycnophyes kielensis*. Maximum projection micrographs obtained with a confocal laser scanning microscope. The nervous system was stained with primary antibodies against serotonin and appears *red*. Cell nuclei where stained with DAPI and appear *blue* together with autofluorescence of the cuticle. In **d** autofluorescence of the cuticle appears *green*. **a** brain of *P. kielensis* with neuropil, flask-shaped somata anterior and posterior of the neuropil, and the ventral nerve cord. **b** 3D reconstruction of **a** showing the dense distribution of nuclei anterior and posterior of the neuropil. **c** Same 3D reconstruction as in **b** but turned 90° horizontally to show the neuropil ring as seen from posterior. **d** Overview of the nervous system with neuropil ring of the brain and ventral nerve cord running ventrally toward the anterior, where it attaches at the ventral nerve cord attachment site, and toward the posterior with a ganglion in the center of segment six behind the pharyngeal bulb. **e** Detail of the neuropil and ventral nerve cord that, when the introvert is inverted, runs from the neuropil toward the anterior and then posteriorly with a ganglion in segment six. *ga* ganglion, *lan* lateral nerve cord, *np* neuropil, *so* somata, *vnc* ventral nerve cord, *vnca* ventral nerve cord attachment site

**Fig. 8:**
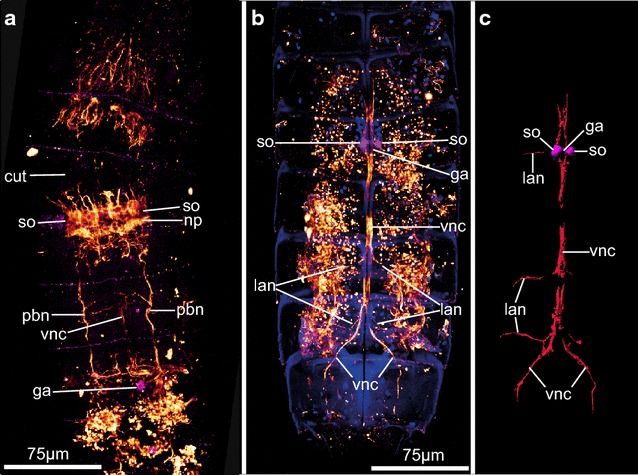
Nervous system of *Pycnophyes kielensis* stained with antibodies against serotonin, α-, and β-tubulin. Maximum projection micrographs obtained with a confocal laser scanning microscope. The nervous system was stained with primary antibodies against serotonin, α-, and β tubulin. Tubulin appears *white-orange*, serotonin appears *pink*. Cell nuclei where stained with DAPI and appear *blue* together with autofluorescence of the cuticle. **a** Overview of the nervous system stained by tubulin and serotonin in the anterior part of *P. kielensis* showing the neuropil ring, pharyngeal bulb nerve cords, and the ganglion in segment six behind the pharyngeal bulb. **b** Overview of the nervous system stained by tubulin and serotonin in the posterior of *P. kielensis* with ganglion in segment six, merged ventral nerve cords, and lateral nerve cords in the posterior segments. *Note* the punctate immunoreactivity is not neuro-specific but most probably the staining of sperm cells that have a microtubule containing tail (axoneme). **c** 3D reconstruction of **b**. *ga* ganglion, *lan* lateral nerve cord, *np* neuropil, *pbn* pharyngeal bulb nerve cord, *so* somata, *vnc* ventral nerve cord

The immunoreactive nervous system of *P. kielensis* consists of a moveable brain, a ventral nerve cord, a ganglion in segment 6, and lateral nerve cords extending from the ventral nerve cord in segments 6–9. The brain of *P. kielensis* comprises a neuropil ring with anteriorly and posteriorly attached flask-shaped somata (Fig. 7a–c). Anterior and posterior of the neuropil ring are densely packed cells that move together with the neuropil when the introvert is moved (Fig. 7a–c). Connected with the neuropil ring is a ventral nerve cord that consists of two merged nerve strands (Figs. 7d, e, 8a, b). The brain is situated at the anterior edge of the pharyngeal bulb and moves together with the introvert. Thus, when the introvert is inverted, the brain lies within segment 4 and the ventral nerve cord runs from there midventrally toward the anterior, where it is attached to the ventral nerve cord attachment site on the midsternal plate. From there, the ventral nerve cord runs midventrally with a prominent ganglion in segment 6 (just posterior of the pharyngeal bulb when the introvert is inverted), and further on toward the posterior until segment 9, where the two merged nerve strands separate and run ventrolaterally into segment 10 (Fig. 8b). Thin lateral nerve cords run laterally from the midventral nerve cord in segments 7–9 (Fig. 8b). Two pharyngeal bulb nerves run laterally from the neuropil along the pharyngeal bulb (Fig. 8a). When the introvert is everted, the brain moves together with the pharyngeal bulb and comes to lie below the primary spinoscalids anterior of segment 1 (Fig. 3g). The ganglion in segment 6 remains stable in its position independent of the position of the introvert and thus the brain.

## Discussion

### Musculature

The musculature of *Pycnophyes kielensis* can functionally be divided into three categories: musculature related to movement, musculature related to feeding and digestion, and musculature related to reproduction. The muscle cells are connected to the cuticle by desmosomes, epidermal intermediate filaments, hemidesmosomes, and thin filaments [1, 58]. One peculiarity of kinorhynchs muscle cells is that they send cell processes toward nerve cells and not the other way around [33]. This is quite unusual as the normal condition in most invertebrates is that nerve cell processes innervate the muscle cells.

### Musculature related to movement

The introvert and mouth cone apparatus in *P. kielensis* are organized like a telescopic cylinder that provides a long output travel from a very compact retracted length. The system is moved by muscles attached to the pharyngeal bulb and probably also by a synchronous contraction of the dorsoventral trunk musculature which builds up pressure [9, 14]. The use of internal pressure, caused by contraction of the dorsoventral musculature to push the introvert out is very likely, as neither the pharyngeal bulb protractors nor the pharyngeal bulb retractors of *P. kielensis* appear very strong. Their function might merely be to arrest the pharyngeal bulb on its way anteriorly. The synchronous contraction of dorsoventral musculature causes the protrusion of the introvert, and the rigidity of the cuticle provides enough force to pull the introvert back again when the dorsoventral muscles relax. The introvert of *P. kielensis* is thus moved passively following the internal body pressure, and forward movement is facilitated by the rigid scalids of the introvert that find the necessary resistance to exert forward moving force in the surrounding sediment.

The longitudinal muscles in the trunk of *P. kielensis* facilitate ventral and dorsal bending. In contrast to cyclorhagid kinorhynchs, which possess oblique muscles that support lateral bending, the species of *Pycnophyes* and *Kinorhynchus* cannot bend laterally [9].

### Musculature related to feeding and digestion

The pharyngeal bulb is the very prominent muscular organ in *P. kielensis*. The feeding apparatus of kinorhynchs functions as a suction pump that sucks in food through the mouth cone by extension of the pharyngeal bulb [62]. In *Pycnophyes dentatus*, the lumen of the pharynx is triradiate, but the outer perimeter is circular [33]. The same organization of the pharyngeal bulb is found in *P. kielensis* with a circular outer layer of musculature (Fig. 2k). The lumen of the investigated specimens in this study appeared folded in orthogonal view.

It is still not fully established what kinorhynchs actually feed on. Diatoms and bacteria have been reported [33, 58, 63]. Given their feeding mode as suction feeders, it is likely that kinorhynchs feed on all the organic material that they suck in, like a tiny vacuum cleaner on the ocean floor. It is also not clear whether the outer oral styles are directly manipulated by the mouth cone muscles, or whether they are merely passively moved and aid in preventing food particles to escape the mouth cone. The presence of nine mouth cone muscles correlates to the nine outer oral styles; however, the mouth cone muscles end in a mouth cone ring muscle and can thus not lie directly within the outer oral styles. Additionally, TEM pictures of a transverse section of the mouth cone of *Pycnophyes greenlandicus* do not show musculature within the oral styles [1]. In *Zelinkaderes floridensis*, each of the outer oral styles can be manipulated by two longitudinal muscles cells [33, 58]. This has been shown by TEM, and it might be that there are muscle cell processes from the mouth cone muscles toward the outer oral styles that were not detectable by CLSM herein.

The midgut muscles have been previously described as segmentally arranged in kinorhynchs, because ‘remnants of circular muscles separate the longitudinal muscles in each segment’ [1]. This is not the case in *P. kielensis* as there is no visible segmentation within the gut musculature. Additionally, the above-mentioned argument cannot hold in any kinorhynch, because the position of the circular muscles changes continuously in relation to the segments, with the movement of the introvert.

### Musculature related to reproduction

Kinorhynchs are gonochoric (have just one of two distinct sexes in any one individual), reproduce sexually and usually show sexual dimorphism [64]. Muscles related to reproduction in *P. kielensis* are found in segments 10–11 and differ between males and females. Females have a dorsal gut opening muscle in segment 10 and males have muscles moving the penile spines in segment 11. It has been suggested that musculature attached to the lateral terminal spines are specializations of the longitudinal musculature [65]. This suggestion was not supported in *Antygomonas* sp. where terminal spine muscles are clearly separated from longitudinal muscles [37], and it is also not the case in *P. kielensis*.

During copulation, the male and female attach to each other with the ventral side of their posterior ends and exchange a spermatophore [64]. The female keeps the sperm in a receptaculum seminis until the eggs are fertilized [12]. Laying of eggs has so far only been reported once for the cyclorhagid kinorhynch *Echinoderes kozloffi* [66]. There, the egg has a diameter of around 50 µm and is enveloped in a ball-shaped mass consisting of mucus and sediment and measuring 260–300 µm in diameter. It is not clear through which opening the eggs are laid.

It has been mentioned that the posterior part of the male gonads is surrounded by a muscle net [9]. This is not the case in *P. kielensis*.

### Musculature in *P. kielensis* compared to other kinorhynchs

The musculature in kinorhynchs has previously been studied using confocal laser scanning microscopy for five cyclorhagid species of the genus *Echinoderes*, *Antygomonas* sp., and *Pycnophyes kielensis* [14, 35–37]. Many of the described muscles are similar among species. The following discussion will focus on the differences.

In contrast to other kinorhynch species, *P. kielensis* does not have circular muscles connecting the placids in the neck region, but has a small transversal neck muscle at the dorsal anterior end of segment 1.

Differences in the trunk musculature between *P. kielensis* and *Echinoderes* and *Antygomonas* are pairs of diagonal muscle bands on left and right lateral sides of the trunk in segments 1–8 (*Echinoderes*) or 1–9 (*Antygomonas*), which are absent in *P. kielensis*. The absence of diagonal muscle bands is a general difference in the musculature between allomalorhagid and cyclorhagid kinorhynchs [9, 12]. Members of *Echinoderes* have dorsoventral muscles in segments 3–11, whereas *P. kielensis* and *Antygomonas* sp. show dorsoventral muscles in segments 1–11. The position of the dorsoventral muscles is similar in all investigated kinorhynchs as it runs from midventral dorsolaterally and attaches to the tergal cuticular plate [14, 37].

*E. spinifurca* has further one pair of ventral muscles and two pairs of dorsal muscles in subdorsal and laterodorsal positions within segments 1–10 [14], whereas *P. kielensis* has two pairs of ventral and dorsal longitudinal muscles within segments 1–9. And two pairs of ventral and one pair of dorsal longitudinal muscles in segment 10.

Some other muscles, previously described in kinorhynchs, could not be confirmed in the same arrangement in *Pycnophyes kielensis*. These are: circular muscles at the base of scalids in rings 06 and 07, head retractor muscles between the base of the head scalids of rings 05 and 06, and posterior trunk segments, mouth cone retractors between the base of the mouth cone and posterior segments, circular muscles connecting the placids in the neck region, and a muscle net around the male gonads [9].

The circular muscles at the base of the scalids in rings 06 and 07 was described by Zelinka [12] and confirmed by TEM in *Echinoderes capitatus* [55]. These circular muscles are either not present in *P. kielensis*, or could not be detected by CLSM herein.

The head retractor muscles (outer retractors) between the base of the head scalids of rings 05 and 06 and posterior trunk segments, as well as the mouth cone retractors (inner retractors) between the base of the mouth cone and posterior segments, have also been described by Zelinka and confirmed by TEM [12, 55].

The mouth cone retractors (inner retractors) between the base of the mouth cone and posterior segments correspond to the pharyngeal bulb’s retractors herein. In the immunostainings, it is clear that the retractors interconnect with the pharyngeal bulb at the bulbs posterior part. In *Echinoderes capitatus*, the retractors seemed to extend along the pharyngeal bulb to insert at the base of the mouth cone instead [55]. In other members of *Echinoderes*, the inner retractors have also been identified as pharynx retractors [14]. The muscles that extend from the pharyngeal bulb toward the mouth cone and end in a muscular mouth cone ring are named mouth cone muscles herein as they are different from, and not interconnected with, the pharyngeal bulb retractors.

The head retractor muscles described by Zelinka correspond to the y-shaped muscles in *P. kielensis* which are situated in between the primary spinoscalids as in members of *Echinoderes* [14]. However, in *Echinoderes* the y-shaped muscles seem to be interconnected with longitudinal proximal retractor muscles, which attach together with trunk musculature at pachycycli of posterior segments [14, 55]. The y-shaped muscles are there thus interpreted as introvert retractors [14]. In *P. kielensis*, it was not possible to find a connection between the y-shaped muscles and trunk musculature and it seems to be physically impossible to have head retractor muscles running from the posterior segments passed the pharyngeal bulb inserting at the base of the scalids when the introvert is everted (see Fig. 3d herein compared to Fig. 1, 2 on plate 18 in Zelinka [28]).

What is particular in kinorhynchs and *P. kielensis* is that the brain is moving together with the introvert and the y-shaped muscles seem to be embedded in the brain when the introvert is everted (Fig. 3g). It has been noted before that muscles are running through the anterior and posterior part of the kinorhynch brain [1]. As the y-shaped muscles turn 180 degrees, when being moved with the introvert, to ʎ-shaped muscles in the everted introvert, it is necessary that at least parts of the anterior brain have to turn together with the y-shaped muscles in order to keep its position in relation to the position of the musculature.

### Musculature in phylogenetic discussion

Musculature on its own is probably not the best character for phylogenetic discussions as the arrangement of muscles is quite flexible and differences can often be found within closely related species, as is the case in *Echinoderes* and other animal groups [14, 67]. There is one feature in the musculature of kinorhynchs which has been considered to be shared with other phyla. That is the introvert musculature which was previously described for *Echinoderes capitatus* as comprising two rings of introvert retractors attached through the collar shaped brain [55]. As a similar organization is found in Priapulida and Loricifera, this character was used as a morphological apomorphy to unite Priapulida, Kinorhycha, and Loricifera into Scalidophora [2]. However, taking a closer look, the muscular arrangement around the collar shaped brain is different in the three groups. As shown herein and previously, the inner set introvert retractors that have been named mouth cone retractors are attached to the posterior part of the pharyngeal bulb and thus not directly attached to the mouth cone [14]. Apart from the muscular arrangement around the brain, the arrangement of the musculature in Priapulida and Loricifera is different when compared to kinorhynchs [68, 69].

In the loriciferan *Nanaloricus* sp., the introvert comprises a net-like muscular arrangement composed of five circular fibers crossed by several thin longitudinal fibers with bifurcated anterior ends [68]. The pre-pharyngeal armature is surrounded by six buccal tube retractors and eight mouth cone retractors [68]. A circular muscle is present in the neck region, and a putative anal sphincter is the posteriormost myoanatomical feature [68]. If one assumes that the last common ancestor of Scalidophora has had an introvert and a digestive system with a pharyngeal bulb, the muscles related to the introvert and pharyngeal bulb, such as the mouth cone retractors and the circular muscle in the neck region, can be considered to be homologous to the respective muscles in cyclorhagid kinorhynchs (there is no circular muscle around the neck in *P. kielensis*). There are no body muscles in *Nanaloricus* sp. that seem to resemble the trunk musculature of kinorhynchs, and it is, however, striking to notice the arrangement of the abdominal transverse muscles in *Nanaloricus* sp. which are arranged in a serial pattern in the posterior part of adult Loricifera [68].

Musculature in the priapulid *Tubiluchus troglodytes* comprises a grid of circular and longitudinal muscle bundles in the body, and additional outer longitudinal muscles in the introvert [69]. The pharynx has protractors and retractors [69–73]. Here, it becomes really difficult to point out which muscles might be homologous with the muscles found in kinorhynchs. This is not to dispute a close relationship between kinorhynchs, priapulids, and loriciferans, it just illustrates that the musculature is a poor character for phylogenetic inferences at higher levels.

### Nervous system

Nervous systems are responsible for the coordination of responses to external and internal stimuli within animals [74]. In kinorhynchs, sensory structures are distributed throughout the body as sensory spots, and concentrated on the head with cephalic sensory organs and sensory cells at the base of the scalids [1, 55, 75]. The immunoreactive nervous system of *P. kielensis* comprising the circumpharyngeal brain, ventral nerve cord, ganglion in segment 6, and lateral and longitudinal nerves along the posterior segments and the pharyngeal bulb does not represent the full nervous system. According to TEM in *Echinoderes capitatus*, *Pycnophyes dentatus*, *P. kielensis*, and *Zelinkaderes floridensis*, the nervous system is composed of a circumpharyngeal brain, 10 longitudinal nerve strands originating from the forebrain, sometimes two commissures per trunk segment, and a mouth cone that is innervated by nine nerves [1, 9, 55]. Especially the nerve cells innervating the mouth cone could not be shown by immunolabeling. The same was found in *Echinoderes spinifurca, Antygomonas paulae,* and *Zelinkaderes brightae* when immunolabeled with antibodies against serotonin [15]. Compared to these chyclorhagid species, the immunolabeled nervous system of *P. kielensis* does not end in a ring in the posterior part of the body but in two separate nerve strands within segment 9 and 10, and the forebrain of *P. kielensis* is not organized in ten distinguishable lobes as is the case in *Echinoderes spinifurca* [1, 15]. Otherwise, the serotonergic nervous system of *P. kielensis* is very similar to the serotonergic nervous systems previously described [15]. The use of α and β tubulin in this study has given more detailed information about the ventral nerve cord and especially the delicate lateral nerves of the posterior segments. The staining of α and β tubulin has also rendered non-neuro-specific signal which was very likely sperm cells (Fig. 8b). FMRFamide stainings have been tried several times, but never rendered a signal in any experiment.

### Nervous system in phylogenetic discussion

In contrast to the musculature, nervous systems have been argued to be good morphological characters for phylogenetic inferences [57, 76]. However, data on scalidophoran nervous systems are still scarce. Based on TEM, in the loriciferans *Nanaloricus mysticus* and *Pliciloricus enigmaticus* the intraepidermal brain contains more than 500 nerve fibers in cross section, surrounds the buccal tube (*N. mysicus*) or the esophagus (*P. enigmaticus*), and is located within the introvert [77, 78]. As in kinorhynchs, the brain is tripartite with an anterior region with dense cells, a neuropil and a dense cell region posterior of the neuropil, and the mouth cone of Loricifera is innervated from the brain [77]. As in some kinorhynchs, the somata, which are attached anteriorly and posteriorly to the neuropil ring, form clusters or lobes [77]. In contrast to kinorhynchs, all scalids of *N. mysticus* are innervated from the brain [79]. Ten longitudinal neurite bundles run from the brain in posterior direction. The two ventral ones are thicker than the others and form the ventral nerve cord [78].

More data are available for priapulids, which have also been investigated by use of immunocytochemistry [41, 80]. Adults of the meiobenthic *Tubiluchus troglodytes* show an intraepidermal nervous system with a circumpharyngeal brain that like in kinorhynchs consists of a central ring of neuropil and both anterior and posterior somata [80]. From the brain emerges an unpaired ventral nerve cord with ganglion like swellings in the neck and caudal region [80]. Longitudinal neurite bundles are running below and between the rows of scalids, and a small cluster of sensory cells is present under each scalid [80]. In contrast to kinorhynchs, the priapulid body wall of the neck and trunk region contains a net of longitudinal and circular neurite bundles that are present in an orthogonal pattern [80]. The intestine is surrounded by a nerve net, and the caudal appendage is innervated from a caudal swelling of the ventral nerve cord and also includes longitudinal and circular nerve bundles in an orthogonal pattern [80].

In adult *Priapulus caudatus,* the circumpharyngeal brain possesses only the anterior part of somata [80, 81]. Hatching larva of *Priapulus caudatus* have a nervous system consisting of a circumoral brain, an unpaired ventral nerve cord, and a caudal ganglion [41]. As in the adults, the circumoral brain of the hatching larva is bipartite, with somata located anterior to the neuropil [41]. A neck ganglion, introvert plexus, and longitudinal neurites become visible after the first moult [41].

Based on the data that are available so far, the organization of the central nervous system comprising a circumpharyngeal brain and a ventral nerve can be regarded as ground pattern within Scalidophora, as this organization is found in all investigated kinorhynchs, loriciferans, and priapulids [15, 41, 77, 80].

### Evolution of organ systems in a phylogenetic context

Scalidophora are grouped together based on the possession of scalids on the introvert [2, 82]. In order to understand the evolution of organ systems within Scalidophora, a stable phylogenetic framework is necessary. Unfortunately, no such consistent framework exists to date. Based on molecular data, Scalidophora group either together with Nematoida (Cycloneuralia) [83], or emerge at the base of Ecdysozoa [84–86]. The problem with most phylogenies is that Loricifera are usually left out and molecular studies including Loricifera find Scalidophora to be a paraphyletic group [87–89]. One recent molecular study found Loricifera to group with Priapulida, but unfortunately did not include kinorhynchs in the analysis [86]. The lorica of Loricifera is a characteristic stiffened cuticular corset which is similar to the lorica of almost all priapulid larvae. Based on this character, Loricifera and Priapulida have been grouped together (Vinctiplicata) with Kinorhynchs as sister group [90, 91]. Because of their cuticle, Cycloneuralia have a quite good fossil record ranging from the Cambrian to modern times, but no fossil kinorhynchs are known to date [92]. The lack of a consistent phylogeny makes it impossible to reconstruct the probable organ system arrangement in the last common ancestor of Scalidophora, other than it very likely had a nervous system comprising a circumpharyngeal brain with somata–neuropil–somata arrangement and a ventral nerve cord.

### Segmentation

Although it is currently impossible to reconstruct the probable organ system arrangement in the last common ancestor of Scalidophora, one can speculate about probable scenarios. Based on the data presented herein, the apparent segmentation of kinorhynchs might be convergent within Ecdysozoa. Arguments, for the independent evolution of segmentation in kinorhynchs from an unsegmented ancestor with continuous musculature in the body, include the continuous trunk musculature in the anterior segments 1–6, continuous pharyngeal bulb protractors and retractors throughout segments 1–6, no sign of segmentation within the digestive system, and the absence of ganglia in most segments. It has been previously shown that the trunk musculature of kinorhynchs is continuous in early developmental stages, which is in contrast to the situation in the segmented arthropods and annelids [35].

## Conclusions

It is suggested that segmentation in kinorhynchs evolved independently from segmentation in other animal groups. This conclusion is supported by continuous trunk musculature in the anterior segments 1–6, continuous pharyngeal bulb protractors and retractors throughout the anterior segments, no sign of segmentation within the digestive system, and the absence of ganglia in most segments. The musculature only shows signs of segmentation in segments 7–10, based on the definition of an anteroposteriorly repeated body unit, as defined by a set of substructures in a specific spatiotemporal correlation.

## Additional file

**Additional file 1.** *Pycnophyes kielensis* moving through sand. Movie showing two specimens of *Pycnophyes kielensis* moving in between sand grains by use of their introvert.

## References

[1] Kristensen RM, Higgins RP, Harrison FW (1991). Kinorhyncha. Microscopic anatomy of invertebrates volume 4: Aschelminthes.

[2] Nielsen C (2012). Animal evolution: interrelationships of the living phyla.

[3] Telford MJ, Bourlat SJ, Economou A, Papillon D, Rota-Stabelli O (2008). The evolution of the Ecdysozoa. Philos Trans R Soc Lond B Biol Sci.

[4] Rota-Stabelli O, Daley AC, Pisani D (2013). Molecular timetrees reveal a Cambrian colonization of land and a new scenario for ecdysozoan evolution. Curr Biol.

[5] Rota-Stabelli O, Campbell L, Brinkmann H, Edgecombe GD, Longhorn SJ, Peterson KJ, Pisani D, Philippe H, Telford MJ (2011). A congruent solution to arthropod phylogeny: phylogenomics, microRNAs and morphology support monophyletic Mandibulata. Proc Biol Sci.

[6] Giribet G, Edgecombe GD (2012). Reevaluating the arthropod tree of life. Annu Rev Entomol.

[7] Borner J, Rehm P, Schill RO, Ebersberger I, Burmester T (2014). A transcriptome approach to ecdysozoan phylogeny. Mol Phylogenet Evol.

[8] Sørensen MV, Dal Zotto M, Rho HS, Herranz M, Sánchez N, Pardos F, Yamasaki H (2015). Phylogeny of Kinorhyncha based on morphology and two molecular loci. PLoS ONE.

[9] Neuhaus B, Schmidt-Rhaesa A (2013). Kinorhyncha (=Echinodera). Handbook of Zoology.

[10] Dujardin F. Observations zoologiques I. Sur un petit animal marin, l’Echinodère, formant un type intermédiaire entre les Crustacés et les Vers. *Ann Sci Naturelles* 1851, 3. Ser. Tome 15:158–160.

[11] Higgins RP, Thiel H, Higgins RP (1988). Kinorhyncha. Introduction to the study of meiofauna.

[12] Zelinka K (1928). Monographie der Echinodera.

[13] Kristensen RM. Kinorhyncha. In: eLS. New York: Wiley; 2001. doi:10.1038/npg.els.0001590/full.

[14] Herranz M, Boyle MJ, Pardos F, Neves RC (2014). Comparative myoanatomy of *Echinoderes* (Kinorhyncha): a comprehensive investigation by CLSM and 3D reconstruction. Front Zool.

[15] Herranz M, Pardos F, Boyle MJ (2013). Comparative morphology of serotonergic-like immunoreactive elements in the central nervous system of kinorhynchs (Kinorhyncha, Cyclorhagida). J Morphol.

[16] Dunn CW, Giribet G, Edgecombe GD, Hejnol A (2014). Animal phylogeny and its evolutionary implications. Annu Rev Ecol Evol Syst.

[17] Vroomans RM, Hogeweg P, Ten Tusscher KH (2016). In silico evo-devo: reconstructing stages in the evolution of animal segmentation. EvoDevo.

[18] Scholtz G (2002). The Articulata hypothesis—or what is a segment?. Org Divers Evol.

[19] Hannibal RL, Patel NH (2013). What is a segment?. EvoDevo.

[20] Davis GK, Patel NH (1999). The origin and evolution of segmentation. Trends Cell Biol.

[21] Starunov VV, Dray N, Belikova EV, Kerner P, Vervoort M, Balavoine G (2015). A metameric origin for the annelid pygidium?. BMC Evol Biol.

[22] De Robertis EM (2008). The molecular ancestry of segmentation mechanisms. Proc Natl Acad Sci USA.

[23] Balavoine G, Adoutte A (2003). The segmented urbilateria: a testable scenario. Integr Comp Biol.

[24] Tautz D (2004). Segmentation. Dev Cell.

[25] Couso JP (2009). Segmentation, metamerism and the Cambrian explosion. Int J Dev Biol.

[26] De Robertis EM (1997). Evolutionary biology. The ancestry of segmentation. Nature.

[27] Stollewerk A, Schoppmeier M, Damen WG (2003). Involvement of Notch and Delta genes in spider segmentation. Nature.

[28] Andrioli LP (2012). Toward new *Drosophila* paradigms. Genesis.

[29] Grimmel J, Dorresteijn AWC, Frobius AC (2016). Formation of body appendages during caudal regeneration in *Platynereis dumerilii*: adaptation of conserved molecular toolsets. EvoDevo.

[30] Neuhaus B, Blasche T (2006). *Fissuroderes*, a new genus of Kinorhyncha (Cyclorhagida) from the deep sea and continental shelf of New Zealand and from the continental shelf of Costa Rica. Zool Anz.

[31] Adrianov AV, Malakhov VV (1994). Kinorhyncha: Structure, development, phylogeny and taxonomy.

[32] Nyholm K-G (1947). Studies in the Echinoderida. Ark Zool.

[33] Neuhaus B (1994). Ultrastructure of alimentary canal and body cavity, ground pattern, and phylogenetic relationships of the Kinorhyncha. Microfauna Mar.

[34] GaOrdóñez D, Pardos F, Benito J (2000). Cuticular structures and epidermal glands of *Echinoderes cantabricus* and *E. hispanicus* (Kinorhyncha, Cyclorhagida) with special reference to their taxonomic value. J Morphol.

[35] Schmidt-Rhaesa A, Rothe BH (2006). Postembryonic development of dorsoventral and longitudinal musculature in *Pycnophyes kielensis* (Kinorhyncha, Homalorhagida). Integr Comp Biol.

[36] Rothe BH, Schmidt-Rhaesa A (2004). Probable development from continuous to segmental longitudinal musculature in *Pycnophyes kielensis* (Kinorhycha, Homalorhagida). Meiofauna Mar.

[37] Müller MC, Schmidt-Rhaesa A (2003). Reconstruction of the muscle system in *Antygomonas* sp. (Kinorhyncha, Cyclorhagida) by means of phalloidin labeling and cLSM. J Morphol.

[38] Lang K (1936). Untersuchungen aus dem Öresund XXI: Einige Kleintiere aus dem Öresund. K Fysiogr Sällsk Handl.

[39] Reimer L (1963). Zur Verbreitung der Kinorhyncha in der mittleren Ostsee. Zool Anz.

[40] Neuhaus B (1988). Ultrastructure of the Protonephridia in *Pycnophyes kielensis* (Kinorhyncha, Homalorhagida). Zoomorphology.

[41] Martin-Duran JM, Wolff GH, Strausfeld NJ, Hejnol A (2016). The larval nervous system of the penis worm *Priapulus caudatus* (Ecdysozoa). Philos Trans R Soc Lond B Biol Sci.

[42] Holland LZ, Carvalho JE, Escriva H, Laudet V, Schubert M, Shimeld SM, Yu J-K (2013). Evolution of bilaterian central nervous systems: a single origin?. EvoDevo.

[43] Pani AM, Mullarkey EE, Aronowicz J, Assimacopoulos S, Grove EA, Lowe CJ (2012). Ancient deuterostome origins of vertebrate brain signalling centres. Nature.

[44] Gerhart J, Lowe C, Kirschner M (2005). Hemichordates and the origin of chordates. Curr Opin Genet Dev.

[45] Moroz LL (2012). Phylogenomics meets neuroscience: how many times might complex brains have evolved?. Acta Biol Hung.

[46] Hirth F (2010). On the origin and evolution of the tripartite brain. Brain Behav Evol.

[47] Arendt D, Denes AS, Jékely G, Tessmar-Raible K (2008). The evolution of nervous system centralization. Philos Trans R Soc Lond B Biol Sci.

[48] Tomer R, Denes AS, Tessmar-Raible K, Arendt D (2010). Profiling by image registration reveals common origin of annelid mushroom bodies and vertebrate pallium. Cell.

[49] Strausfeld NJ, Hirth F (2013). Deep homology of arthropod central complex and vertebrate basal ganglia. Science.

[50] Strausfeld NJ (2010). Brain homology: Dohrn of a new era?. Brain Behav Evol.

[51] Hirth F, Kammermeier L, Frei E, Walldorf U, Noll M, Reichert H (2003). An urbilaterian origin of the tripartite brain: developmental genetic insights from *Drosophila*. Development.

[52] Sen S, Reichert H, VijayRaghavan K (2013). Conserved roles of *ems/Emx* and *otd/Otx* genes in olfactory and visual system development in *Drosophila* and mouse. Open Biol.

[53] Bailly X, Reichert H, Hartenstein V (2013). The urbilaterian brain revisited: novel insights into old questions from new flatworm clades. Dev Genes Evol.

[54] Ryan JF, Pang K, Schnitzler CE, Nguyen AD, Moreland RT, Simmons DK, Koch BJ, Francis WR, Havlak P, Program NCS (2013). The genome of the ctenophore *Mnemiopsis leidyi* and its implications for cell type evolution. Science.

[55] Nebelsick M (1993). Introvert, mouth cone, and nervous system of *Echinoderes capitatus* (Kinorhyncha, Cyclorhagida) and implications for the phylogenetic relationships of Kinorhyncha. Zoomorphology.

[56] Adrianov AV, Malakhov VV (1991). The nervous system of Cephalorhyncha, Kinorhyncha. Zool Zh.

[57] Richter S, Loesel R, Purschke G, Schmidt-Rhaesa A, Scholtz G, Stach T, Vogt L, Wanninger A, Brenneis G, Doring C (2010). Invertebrate neurophylogeny: suggested terms and definitions for a neuroanatomical glossary. Front Zool.

[58] Neuhaus B, Higgins RP (2002). Ultrastructure, biology, and phylogenetic relationships of Kinorhyncha. Integr Comp Biol.

[59] Ahlrichs WH (1995). Ultrastruktur und Phylogenie von Seison nebaliae (Grube 1859) und Seison annulatus (Claus 1876).

[60] Higgins RP (1964). A method for meiobenthic invertebrate collection. Am Zool.

[61] Schneider CA, Rasband WS, Eliceiri KW (2012). NIH Image to ImageJ: 25 years of image analysis. Nat Meth.

[62] Nielsen C (2013). The triradiate sucking pharynx in animal phylogeny. Invertebr Biol.

[63] Adrianov AV, Malakhov VV, Yushin VV (1992). Intracellular endosymbionts and parasites in the gut epithelium of kinorhynchs (Cephalorhyncha, Kinorhyncha). Biol Morya Vlad.

[64] Neuhaus B, Knobil E, Neill JD (1999). Kinorhyncha. Encyclopedia of reproduction.

[65] Hyman LH. Class Kinorhyncha. In: Hyman LH, editor. The invertebrates. Volume 3. New York: McGraw-Hill; 1951. p. 170–183.

[66] Kozloff EN (2007). Stages of development, from first cleavage to hatching, of an *Echinoderes* (Phylum Kinorhyncha: class Cyclorhagida). Cah Biol Mar.

[67] Altenburger A, Wanninger A (2009). Comparative larval myogenesis and adult myoanatomy of the rhynchonelliform (articulate) brachiopods *Argyrotheca cordata*, *A. cistellula*, and *Terebratalia transversa*. Front Zool.

[68] Neves RC, Bailly X, Leasi F, Reichert H, Sorensen MV, Kristensen RM (2013). A complete three-dimensional reconstruction of the myoanatomy of Loricifera: comparative morphology of an adult and a Higgins larva stage. Front Zool.

[69] Rothe BH, Schmidt-Rhaesa A, Todaro MA (2006). The general muscular architecture in *Tubiluchus troglodytes* (Priapulida). Meiofauna Mar.

[70] Lemburg C. Ultrastrukturelle Untersuchungen an den Larven von Halicryptus spinulosus und Priapulus caudatus. Hypothesen zur Phylogenie der Priapulida und deren Bedeutung für die Evolution der Nemanthelminthes. Göttingen: Cuvillier Verlag Göttingen; 1999.

[71] Storch V, Higgins RP, Malakhov VV, Adrianov AV (1994). Microscopic anatomy and ultrastructure of the introvert of *Priapulus caudatus* and *P. tuberculatospinosus* (Priapulida). J Morphol.

[72] Storch V, Higgins RP, Morse MP (1989). Ultrastructure of the body wall of *Meiopriapulus fijiensis* (Priapulida). T Am Microsc Soc.

[73] Storch V, Higgins RP, Rumohr H (1990). Ultrastructure of introvert and pharynx of *Halicryptus spinulosus* (Priapulida). J Morphol.

[74] Schmidt-Rhaesa A. Nervous system. In: Schmidt-Rhaesa A, editor. The evolution of organ systems. Oxford: Oxford University Press; 2007. p. 95–117.

[75] Neuhaus B (1997). Ultrastructure of the cephalic sensory organs of adult *Pycnophyes dentatus* and of the first juvenile stage of *P. kielensis* (Kinorhyncha, Homalorhagida). Zoomorphology.

[76] Harzsch S (2006). Neurophylogeny: architecture of the nervous system and a fresh view on arthropod phyologeny. Integr Comp Biol.

[77] Bang-Berthelsen IH, Schmidt-Rhaesa A, Kristensen RM, Schmidt-Rhaesa A (2013). Loricifera. Handbook of Zoology.

[78] Kristensen RM, Harrison FW, Ruppert EE (1991). Loricifera. Microscopic anatomy of invertebrates.

[79] Kristensen RM (1983). Loricifera, a new phylum with Aschelminthes characters from the meiobenthos. Z Zool Syst Evol.

[80] Rothe BH, Schmidt-Rhaesa A (2010). Structure of the nervous system in *Tubiluchus troglodytes* (Priapulida). Invertebr Biol.

[81] Adrianov AV, Malakhov VV (1996). Priapulida: structure, development, phylogeny and classification.

[82] Minelli A (2008). Perspectives in animal phylogeny and evolution.

[83] Dunn CW, Hejnol A, Matus DQ, Pang K, Browne WE, Smith SA, Seaver E, Rouse GW, Obst M, Edgecombe GD (2008). Broad phylogenomic sampling improves resolution of the animal tree of life. Nature.

[84] Campbell LI, Rota-Stabelli O, Edgecombe GD, Marchioro T, Longhorn SJ, Telford MJ, Philippe H, Rebecchi L, Peterson KJ, Pisani D (2011). MicroRNAs and phylogenomics resolve the relationships of Tardigrada and suggest that velvet worms are the sister group of Arthropoda. Proc Natl Acad Sci USA.

[85] Pisani D, Carton R, Campbell LI, Akanni WA, Mulville E, Rota-Stabelli O, Minelli A, Boxshall G, Fusco G (2013). An overview of arthropod genomics, mitogenomics, and the evolutionary origins of the arthropod proteome. Arthropod biology and evolution: molecules, development, morphology.

[86] Laumer CE, Bekkouche N, Kerbl A, Goetz F, Neves RC, Sorensen MV, Kristensen RM, Hejnol A, Dunn CW, Giribet G, Worsaae K (2015). Spiralian phylogeny informs the evolution of microscopic lineages. Curr Biol.

[87] Yamasaki H, Fujimoto S, Miyazaki K (2015). Phylogenetic position of Loricifera inferred from nearly complete 18S and 28S rRNA gene sequences. Zool Lett.

[88] Park J-K, Rho HS, Kristensen RM, Kim W, Giribet G (2006). First molecular data on the phylum Loricifera—an investigation into the phylogeny of Ecdysozoa with emphasis on the positions of Loricifera and Priapulida. Zool Sci.

[89] Sørensen MV, Hebsgaard MB, Heiner I, Glenner H, Willerslev E, Kristensen RM (2008). New data from an enigmatic phylum: evidence from molecular sequence data supports a sister-group relationship between Loricifera and Nematomorpha. J Zool Syst Evol Res.

[90] Peel JS (2010). A corset-like fossil from the Cambrian Sirius Passet Lagerstätte of North Greenland and its implications for cycloneuralian evolution. J Paleont.

[91] Peel JS, Stein M, Kristensen RM (2013). Life cycle and morphology of a cambrian stem-lineage loriciferan. PLoS ONE.

[92] Maas A, Schmidt-Rhaesa A (2013). Gastrotricha, Cycloneuralia and Gnathifera: The Fossil Record. Handbook of Zoology.

